# Risk of hypoglycemia associated with repaglinide combined with clopidogrel, a retrospective cohort study

**DOI:** 10.1186/s40780-020-00159-7

**Published:** 2020-03-18

**Authors:** Yuuki Akagi, Akiko Iketaki, Haruna Kimura, Yuki Matsudaira, Takami Yoshida, Takahiro Nishimura, Yohei Kawano, Yasunari Mano, Erina Shigematsu, Makoto Ujihara

**Affiliations:** 1 Department of Pharmacy, National Hospital Organization Yokohama Medical Center, 3-60-2 Harajuku, Totsuka, Yokohama, Kanagawa 245-8575 Japan; 2 Department of Pharmacy, National Cancer Center Hospital East, 6-5-1 Kashiwanoha, Kashiwa, Chiba, 277-8577 Japan; 3 Faculty of Pharmaceutical Sciences, Tokyo University of Science, 2641 Yamazaki, Noda, Chiba, 278-8510 Japan; 4 Department of Clinical Laboratory, National Hospital Organization Yokohama Medical Center, 3-60-2 Harajuku, Totsuka, Yokohama, Kanagawa 245-8575 Japan; 5 Department of Diabetes Endocrinology, National Hospital Organization Yokohama Medical Center, 3-60-2 Harajuku, Totsuka, Yokohama, Kanagawa 245-8575 Japan

**Keywords:** Repaglinide, Clopidogrel, Drug-drug interaction, Hypoglycemia, Cytochrome P450 2C8, Mitiglinide

## Abstract

**Background:**

Repaglinide is widely prescribed to reduce postprandial hyperglycemia and elevated glycated hemoglobin (HbA1c) levels associated with type 2 diabetes, and clopidogrel is a thienopyridine antiplatelet agent and widely used in cardiovascular and cerebrovascular diseases. It has been suggested that the concomitant use of repaglinide with clopidogrel may inhibit repaglinide metabolism, because repaglinide is a substrate of cytochrome P450 2C8 (CYP2C8) and the main metabolite of clopidogrel acyl-β-D-glucuronide inhibits CYP2C8 activity. In this study, we retrospectively investigated the effect of clopidogrel with repaglinide on plasma glucose and the risk of hypoglycemia associated with the combination of both drugs.

**Method:**

Patients were taking clopidogrel (75 mg/day) and started taking glinide (1.5 mg/day repaglinide or 30 mg/day mitiglinide) for the first time from April 2012 to March 2017. We targeted subjects who were hospitalized at the start of glinide and whose preprandial plasma glucose was measured by a nurse. The glucose levels were collected for up to 5 days before and after the glinide start date.

**Results:**

Average fasting plasma glucose levels (before breakfast) in the repaglinide and clopidogrel group before and after starting repaglinide were 180.1±35.5 and 136.5 ± 44.1 mg/dL, with a mean decrease of 43.6 ± 33.6 mg/dL. In contrast, there was only a moderate decrease of 11.6 ± 30.0 mg/dL in the mitiglinide and clopidogrel group. Minimum plasma glucose levels in the repaglinide and clopidogrel group before and after starting repaglinide were 145.2 ± 42.9 and 93.3 ± 36.3 mg/dL, respectively. Decrease in minimum levels after starting glinide in the repaglinide and clopidogrel group (51.9 ± 47.5 mg/dL) was more significant than those in the mitiglinide and clopidogrel group (only 2.1 ± 29.1 mg/dL), and the repaglinide group (without clopidogrel, 15.5 ± 20.0 mg/dL). Hypoglycemia was observed in 6 of 15 patients in the repaglinide and clopidogrel group, but only 1 of 15 patients in the mitiglinide and clopidogrel group, and no patients in the repaglinide group.

**Conclusion:**

These findings indicate that minimum plasma glucose levels were significantly decreased in patients taking repaglinide and clopidogrel. Considering the risk of hypoglycemia associated with taking repaglinide and clopidogrel, when a glinide is required in patients taking clopidogrel, mitiglinide may be a better choice.

## Introduction

Repaglinide acts on sulfonylurea (SU) receptors on pancreatic β cells to promote insulin secretion, and suppresses postprandial elevation of plasma glucose [1]. It was the third glinide approved in Japan (in 2011) after nateglinide and mitiglinide. Attention needs to be paid to hypoglycemia in patients taking glinides. However, it is thought to occur less frequently than in patients taking sulfonylurea (SU) drugs [2]. Glinides are widely prescribed to reduce postprandial hyperglycemia and elevated glycated hemoglobin (HbA1c) levels associated with type 2 diabetes, either as a single agent or in combination with an α-glucosidase inhibitor, biguanide, dipeptidyl peptidase-4 inhibitor, etc. [3, 4].

Clopidogrel is a thienopyridine antiplatelet agent that is widely used to treat patients undergoing stent placement as a percutaneous coronary intervention for acute coronary syndrome, stable angina, or an old myocardial infarction, and ischemic stroke or established peripheral arterial disease. Clopidogrel is metabolized by cytochrome P450 (CYP) 2C19, and the main metabolite of clopidogrel acyl-β-D-glucuronide has been shown to inhibit CYP2C8 activity [5]. It is thought that concomitant administration of clopidogrel may inhibit repaglinide metabolism, because repaglinide is a substrate of CYP2C8 [6].

In a study of the combined administration of clopidogrel (300 mg on the first day and 75 mg on the second to third day) and repaglinide (0.25 mg as a single dose), the geometric mean area under the concentration-time curve (AUC_0-∞_) of repaglinide was increased 3.9–5.1 fold, and the peak plasma concentration (C_max_) was increased 2.0–2.5 fold when compared with the control [7]. Thus, repaglinide may increase the risk of hypoglycemia in patients taking clopidogrel, and the concomitant use of both agents has been contraindicated since 2015 in Canada [8]. In Japan, this interaction is described in the precautions for co-administration on medication package inserts [3]. However, this was based on the findings of a study of healthy adults in Finland, and there have been only few case reports [9] and only one study of healthy volunteers [5] in Japan. There are likely to be some cases in which repaglinide and clopidogrel are prescribed together.

In this study, we retrospectively investigated the effect of clopidogrel on plasma glucose in patients taking repaglinide and evaluated the risk of hypoglycemia in patients taking both drugs. We also discussed how to avoid this interaction.

## Subjects and methods

### Patients

**Fig. 1:**
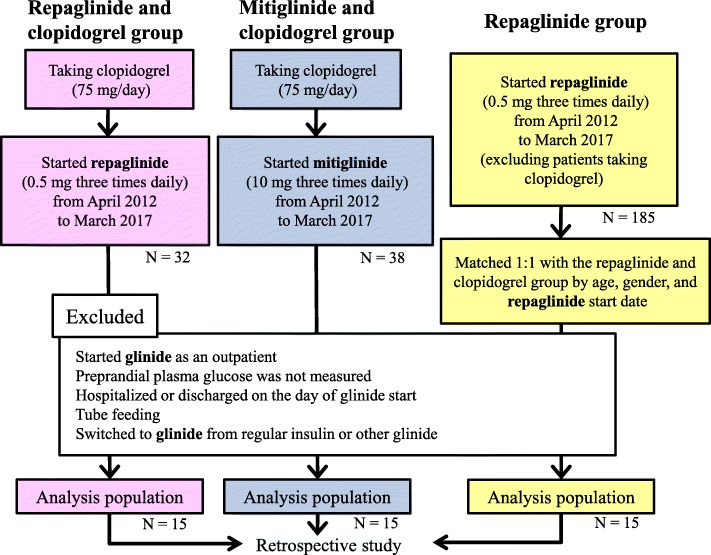
Study flow chart

Patients in the repaglinide and clopidogrel group were taking clopidogrel (75 mg/day) and started taking repaglinide (1.5 mg/day) for the first time from April 2012 to March 2017 at National Hospital Organization Yokohama Medical Center. Patients in the mitiglinide and clopidogrel group were taking clopidogrel (75 mg/day) and started taking mitiglinide (30 mg/day) for the first time during the same time period. Furthermore, we matched patients in the repaglinide and clopidogrel group to other patients who started taking repaglinide (1.5 mg/day, without clopidogrel) during the same period (1:1 by age [the same or nearest age], gender, and start date). Matched cases were analyzed as the repaglinide group (Fig. 1). We targeted subjects who were hospitalized at the start of glinide and whose preprandial plasma glucose was measured by a nurse. Therefore, if the patient started taking glinide as an outpatient, if fasting plasma glucose was not measured around the start of glinide, or if the patient was hospitalized or discharged on the day glinide was started, they were excluded because plasma glucose could not be used as an objective indicator. Patients who received tube feeding or switched from regular insulin or other glinide to the glinide were also excluded from the analysis. This study was approved by the Ethics Committee of National Hospital Organization Yokohama Medical Center (approval number 28–32).

### Collected data

The following data were collected: age (on the day glinide was started), sex, the date repaglinide or mitiglinide was started, body mass index (BMI), HbA1c, concomitant agent use, serum creatinine, liver function (aspartate transaminase [AST], alanine transaminase [ALT], and total bilirubin [T-Bil]), and calorie intake (calculated from the provided calorie and intake ratio). The data points nearest to the glinide starting day were used for BMI, HbA1c, serum creatinine, and liver function.

In addition to oral hypoglycemic drugs, insulin, and glucagon-like peptide-1 (GLP-1) agonists, other concomitant agents that may enhance the hypoglycemic action of repaglinide or mitiglinide (described in package insert in Japan [3, 4]) were investigated, including β-blockers, monoamine oxidase inhibitors, salicylic acid, anabolic steroids, tetracycline antibiotics, cyclosporine, deferasirox, sulfamethoxazole trimethoprim combination, and clofibrate. If any of the above drugs were used, the case was marked as “concomitant agent use.”

### Plasma glucose

**Fig. 2:**
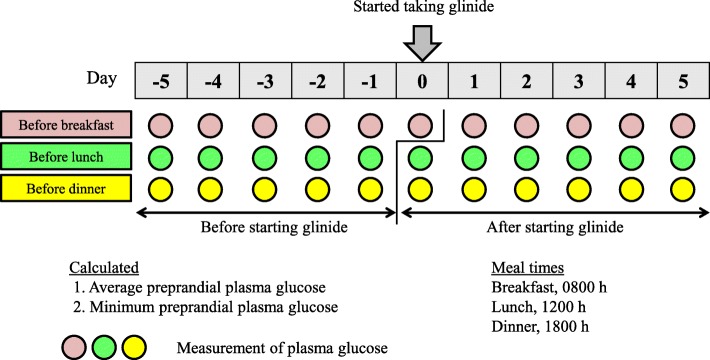
Analysis of plasma glucose levels before and after starting glinide. Examples are shown (started taking glinide from “before breakfast”)

Preprandial plasma glucose levels (30 min before a meal) were collected for up to 5 days before and after the glinide start date (Fig. 2). Plasma glucose was measured by enzyme electrode method (FreeStyle Precision Pro, blood glucose monitoring system for the hospital). In this study, hypoglycemia was defined as a plasma glucose level less than 70 mg/dL. Plasma glucose before taking repaglinide or mitiglinide for the first time was used as the “before starting glinide” data, and plasma glucose after taking glinide was used as the “after starting glinide” data. Average plasma glucose levels before breakfast, lunch, and dinner, and the decreased amplitude of glycemic excursions were calculated. In addition, the difference between the lowest plasma glucose levels before and after the start of glinide was also calculated.

### Statistical analysis

One-way analysis of variance was used for quantitative variables (age, BMI, HbA1c, serum creatinine, and calorie intake), Fisher’s exact test was used for qualitative variables with values in any of cells of 5 or below (liver function and patients with hypoglycemia), and Chi-square test was used for other qualitative variables such as sex and concomitant agent, to evaluate differences in the clinical characteristics of patients between 3 groups. Tukey’s honestly significant difference test and Bonferroni’s collection were used to analyze the decreases in glycemic levels before breakfast, lunch, and dinner, and the difference between the lowest plasma glucose levels before and after starting glinide. The numbers of patients with hypoglycemia symptoms were analyzed by Fisher’s exact test using Bonferroni’s adjusted significance level. All statistical analyses were carried out using SPSS Statistics version 23.0 (IBM Japan, Ltd.), and *p* values less than 5% were considered to be significant.

## Results

### Glycemic excursions

During the study period, there were 32 patients who were taking clopidogrel and started taking repaglinide for the first time. Of these, 17 were excluded based on the study criteria. Therefore, 15 patients were analyzed as the repaglinide and clopidogrel group. In addition, there were 38 patients taking clopidogrel who started taking mitiglinide for the first time. However, 23 of these patients were excluded, and 15 subjects were analyzed as the mitiglinide and clopidogrel group. There were 180 patients who started taking repaglinide but were not in the repaglinide and clopidogrel group, and 15 of these were matched to the patients in the repaglinide and clopidogrel group and were analyzed as the repaglinide group (Table 1).

**Table 1:** Clinical characteristics of study patients

Characteristics	Repaglinide and clopidogrel group	Mitiglinide and clopidogrel group	Repaglinide group	*p* value
	(*N* = 15)	(*N* = 15)	(*N* = 15)	
Age	75.6 ± 12.2	70.4 ± 11.5	76.1 ± 10.9	0.332*
Male (%)	10 (66.7%)	12 (80.0%)	10 (66.7%)	0.649***
BMI	23.6 ± 3.2	24.4 ± 2.9	23.2 ± 3.9	0.598*
HbA1c	9.3 ± 2.0	9.5 ± 2.0	9.7 ± 2.6	0.851*
Serum creatinine	0.84 ± 0.32	0.80 ± 0.16	0.76 ± 0.16	0.594*
Liver function (Grade 1 [CTCAE ver.4.0]: AST, ALT, or T-Bil)	4 (26.7%)	5 (33.3%)	3 (20.0%)	0.912**
Calorie intake (kcal)	1325.9 ± 283.3	1497.0 ± 247.5	1517.2 ± 168.7	0.065*
Concomitant agent use (oral hypoglycemic drugs, insulin, GLP-1 agonists, and/or agents which may enhance the hypoglycemic action of repaglinide or mitiglinide, other than the above)	10 (67.7%)	9 (60.0%)	9 (60.0%)	0.910***

Mean ± S.D., * One-way analysis of variance, ** Fisher’s exact test, *** Chi-square test

The average plasma glucose levels before breakfast in the repaglinide and clopidogrel group before and after the repaglinide start date were 180.1 ± 35.5 and 136.5 ± 44.1 mg/dL, respectively, and the mean decrease in plasma glucose was 43.6 ± 33.6 mg/dL (Table 2). The corresponding mean decreases in plasma glucose before lunch and dinner were 50.0 ± 36.6 and 61.6 ± 49.3 mg/dL, respectively. In the mitiglinide and clopidogrel group, preprandial plasma glucose levels decreased by 11.6 ± 30.0 (breakfast), 55.8 ± 43.3 (lunch), and 24.6 ± 45.0 mg/dL (dinner), which were relatively moderate declines compared to those in the repaglinide and clopidogrel group. In the repaglinide group, preprandial plasma glucose levels decreased by 23.2 ± 23.7 (breakfast), 27.4 ± 38.9 (lunch), and 42.5 ± 38.9 mg/dL (dinner).

**Table 2:** Preprandial plasma glucose levels

Preprandial plasma glucose	Repaglinide and clopidogrel group	Mitiglinide and clopidogrel group	Repaglinide group
	(*N* = 15)	(*N* = 15)	(*N* = 15)
Breakfast			
Before starting glinide	180.1 ± 35.5	161.8 ± 38.0	151.0 ± 20.9
After starting glinide	136.5 ± 44.1	150.1 ± 41.3	127.8 ± 33.0
Decrease in the amplitude of glycemic excursions	43.6 ± 33.6 *	11.6 ± 30.0	23.2 ± 23.7
Lunch			
Before starting glinide	228.2 ± 48.6	231.7 ± 48.7	196.1 ± 32.3
After starting glinide	178.8 ± 69.8	177.2 ± 53.9	168.7 ± 37.2
Decrease in the amplitude of glycemic excursions	50.0 ± 36.6	55.8 ± 43.3	27.4 ± 38.9
Dinner			
Before starting glinide	193.3 ± 54.5	190.5 ± 47.6	186.8 ± 56.5
After starting glinide	131.6 ± 43.4	165.9 ± 44.6	144.3 ± 52.8
Decrease in the amplitude of glycemic excursions	61.6 ± 49.3	24.6 ± 45.0	42.5 ± 38.9

Mean ± S.D., * *p* < 0.05 for the “repaglinide and clopidogrel group” versus the “mitiglinide and clopidogrel group”

The minimum plasma glucose levels in the repaglinide and clopidogrel group before and after the starting repaglinide were 145.2 ± 42.9 and 93.3 ± 36.3 mg/dL, respectively, with a decrease of 51.9 ± 47.5 mg/dL (Table 3). The decreased amplitude was 2.1 ± 29.1 mg/dL in the mitiglinide and clopidogrel group and 15.5 ± 20.0 mg/dL in the repaglinide group. Decrease in minimum levels after starting glinide in the repaglinide and clopidogrel group was more significant than those in the other 2 groups. As shown in Table 1, no significant differences were observed in age, sex, BMI, HbA1c, serum creatinine, calorie intake, and concomitant agent use. Liver function (AST, ALT, and T-Bil) in all patients was either normal or mildly impaired (grade 1 according to the Common Terminology Criteria for Adverse Events [CTCAE] ver. 4.0).

**Table 3:** Decrease in minimum plasma glucose levels

Minimum plasma glucose	Repaglinide and clopidogrel group	Mitiglinide and clopidogrel group	Repaglinide group
	(*N* = 15)	(*N* = 15)	(*N* = 15)
Before starting glinide	145.2 ± 42.9	133.5 ± 33.5	113.6 ± 23.5
After starting glinide	93.3 ± 36.3	131.4 ± 42.9	98.1 ± 21.8
Decrease in minimum levels	51.9 ± 47.5 *	2.1 ± 29.1	15.5 ± 20.0

Mean ± S.D., * *p* < 0.05 for the “repaglinide and clopidogrel group” versus the “mitiglinide and clopidogrel group”, and the “repaglinide and clopidogrel group” versus the “repaglinide group”

### Hypoglycemia

Hypoglycemia (plasma glucose: 43–68 mg/dL) was observed in 6 of 15 patients in the repaglinide and clopidogrel group, significantly more patients compared to the repaglinide group; however it was observed in only 1 of 15 patients (plasma glucose: 52 mg/dL) in the mitiglinide and clopidogrel group.

The minimum plasma glucose levels measured 5 days before the combination start date were less than 150 mg/dL in 10 cases in both the “repaglinide and clopidogrel” and “mitiglinide and clopidogrel” groups; and among these patients, 6 in the repaglinide and clopidogrel group and 1 in the mitiglinide and clopidogrel group developed hypoglycemia (Table 4). In contrast, among the 5 patients in both groups with minimum plasma glucose levels of 150 mg/dL or higher, no hypoglycemia was observed.

**Table 4:** Number of patients with hypoglycemia

Number of patients	Repaglinide and clopidogrel group	Mitiglinide and clopidogrel group	Repaglinide group
	(*N* = 15)	(*N* = 15)	(*N* = 15)
Patients with hypoglycemia	6 (40.0%)*	1 (6.7%)	0 (0.0%)

Differences of hypoglycemia incidence rates in “repaglinide group versus repaglinide and clopidogrel group” and “repaglinide and clopidogrel group versus mitiglinide and clopidogrel group” were analyzed by Fisher’s exact test using Bonferroni’s adjusted significant level

*p < 0.05 for the repaglinide group versus repaglinide and clopidogrel group

## Discussion

In the treatment of diabetes, it is very important to maintain good plasma glucose control, without hypoglycemia. In recent years, several large-scale clinical trials have reported that severe hypoglycemia as a result of intensive glycemic control is associated with the onset of adverse events, such as cardiovascular events and dementia [10, 11]. According to the Japan Diabetes Society, a lower HbA1c limit is set for older patients with diabetes if they are prescribed a drug, such as insulin, an SU drug, or a glinide, which may cause severe hypoglycemia [12]. Thus, safer treatment is proposed to prevent hypoglycemia.

In this study, preprandial plasma glucose in the repaglinide and clopidogrel group was decreased by 40–60 mg/dL after starting repaglinide, and this same tendency to decrease was observed in the repaglinide group. Glinide significantly improved plasma glucose levels from 1 to 2 h after a meal; but the reduction in fasting plasma glucose was relatively small [1]. In the mitiglinide and clopidogrel group, the reduction in plasma glucose before breakfast was lower than that in the repaglinide and clopidogrel group. This seemed to be related to the fact that preprandial plasma glucose in the morning was measured more than 12 h after the last meal, whereas when preprandial plasma glucose was measured at noon (lunch) or in the evening (dinner), it was only about 4–6 h since the last meal (Fig. 2).

To evaluate the risk of hypoglycemia, it is necessary to consider not only the average plasma glucose level and its variation but also the lowest level. In the repaglinide and clopidogrel group, the minimum blood glucose level was significantly reduced, by about 50 mg/dL, after starting repaglinide. In addition, hypoglycemia was observed in 6 of 15 patients, and repaglinide was discontinued in all these cases. Renal function in all 6 of these cases was normal or mildly impaired, but 5 of these patients were over 65 years old, and it was presumed that this excessive reduction in plasma glucose might be caused by the interaction between repaglinide and clopidogrel. In addition, one case developed hypoglycemia after the analysis period, and one case with reduced plasma glucose (to 75 mg/dL). However, no hypoglycemia was observed in the repaglinide group. In the mitiglinide and clopidogrel group, hypoglycemia was observed in only 1 patient. This patient was a female in her 80s with moderately impaired renal function who was considered to be at high risk for hypoglycemia. The metabolism of mitiglinide is different from that of repaglinide, and glucuronidation is produced by other enzymes (UDP- glucuronosyltransferases 1A3 and 2B7) [13, 14].

To consider how to avoid interaction between repaglinide and clopidogrel, patients taking mitiglinide plus clopidogrel were collected in this study. This group has a similar patient background besides diabetes, such as the presence of cerebrovascular and cardiovascular disease. Although no study has directly compared the effects of repaglinide and mitiglinide, significant reductions in HbA1c and fasting plasma glucose were observed in a report of patients who switched from mitiglinide (30 mg/day) to repaglinide (1.5 mg/day) [15], and it was presumed that the hypoglycemic effect of repaglinide (1.5 mg/day) was stronger than that of mitiglinide (30 mg/day). However, hypoglycemia was not observed in either glinide [15]. Therefore, it was postulated that the difference in the effects of the glinides themselves has little influence.

In earlier studies, risk of hypoglycemia was reported in concomitant use of repaglinide and clopidogrel using real world data of Taiwan [16]. In our study, as shown before, hypoglycemia was defined as a plasma glucose level less than 70 mg/dL in electronic medical record, as opposed to diagnosis code in the real world database. It was considered that more accurate analysis could be performed by evaluating plasma glucose levels. Although few studies have focused on the interaction between repaglinide and clopidogrel in Japan, there have been case reports of persistent and severe hypoglycemia in elderly patients with renal dysfunction [9]. In the patients with hypoglycemia in our study, the minimum plasma glucose levels before starting the combination were 94–141 mg/dL. If plasma glucose is less than 150 mg/dL, the risk of hypoglycemia may be higher when using both drugs. After revision of the medication package insert in 2016, repaglinide has not been prescribed for patients taking clopidogrel in the Diabetes and Endocrinology department of our hospital. In accordance with our results, when a glinide is needed for patients taking clopidogrel, mitiglinide is selected.

There are some limitations in this study. First, this was a retrospective study, and any influence of unknown and unpredictable background factors cannot be excluded. Second, CYP2C8 gene polymorphisms were not investigated. It has been reported that the AUC_0-∞_ and C_max_ of a single 2 mg oral dose of repaglinide were 105.9 ng∙h/mL and 60.3 ng/mL, respectively, for wild type (*1/*1) CYP2C8, and 72.4 ng∙h/mL and 38.5 ng/mL for *3/*3 CYP2C8, respectively, and the plasma concentration decreased due to metabolic activation [17]. However, the mean and maximum amplitudes of the glycemic excursions were 0.42 and 1.62 mM for *1/*1, and were 0.50 and 1.48 mM for *3/*3, respectively. Thus, the influence of polymorphism was small, and it is not a problem clinically. Third, plasma glucose levels were collected for up to only 5 days after the start of glinide. The following points can be given as reasons; neither repaglinide nor mitiglinide were considered to have a cumulative effect due to continuous administration [18, 19], and it was considered that continuous preprandial plasma glucose levels for patients during hospitalization were one of the best objective and important indicators to evaluate hypoglycemia. In addition, our analysis consisted of only 15 cases in each group in a single hospital. Although this was a small number of patients, the finding was remarkable because a large increase in the risk of hypoglycemia was observed.

## Conclusion

The results of this study indicate that plasma glucose levels before a meal were greatly decreased, and the minimum plasma glucose levels were significantly decreased in patients taking repaglinide with clopidogrel. Based on the observed risk of hypoglycemia, when a glinide is needed for patients taking clopidogrel, mitiglinide may be a better choice.

## Data Availability

The datasets used and analyzed during the current study are available from the corresponding author on reasonable request.

## Abbreviations

ALT: Alanine transaminase
AST: Aspartate transaminase
AUC_0-∞_: Area under the concentration-time curve
BMI: Body mass index
C_max_: Peak plasma concentration
CTCAE: Common Terminology Criteria for Adverse Events
CYP: Cytochrome P450
GLP-1: Glucagon-like peptide-1
HbA1c: Glycated hemoglobin
SU: Sulfonylurea
T-Bil: Total bilirubin
